# Between Drs. Baldwin and Nott, in Relation to the Next Meeting of the Am. Medical Association

**Published:** 1869-04-15

**Authors:** 


Dr. Baldwin desires the medical journals of the country to publish these letters in full or to give an analysis of them in April. E. S. GAILLARD.

### *Advance Sheets of the Richmond and Louisville Medical Journal for April, 1869.*

[LETTER I.]

MONTGOMERY, ALA., March 15, 1869.

DR. E. S. GAILLORD, EDITOR RICHMOND AND LOUISVILLE MEDICAL JOURNAL:

MY DEAR SIR:—I send you this letter and the inclosed correspondence between Dr. J. C. Nott and myself, for publication in your journal.

You must pardon me, dear doctor, for the personal allusion contained in this correspondence to yourself. From the fact that you were an active participant in the late war, and suffered deeply by its results, and from the additional fact that you have occupied a prominent position in the medical profession before and since the war, I thought I might take the liberty of referring to you as a true representative of the professional sentiment of the South.

For the same reason I addressed a communication to Dr. Nott, (formerly of Mobile, now of New York,) whom it is well known, was a staunch adherent of the Confederate cause; who, at the advanced age of sixty years, gave up his professorship in a college to which he was devoted and of which he was the founder; relinquished his large and lucrative practice and neglected his then ample fortune to take a commission in the army of the South; serving in hospitals, in camp, on the march, in the front or wherever he was ordered, with all the devotion and faithfulness of his enthusiastic and honest nature. He had but three children, all sons; one lost an arm in infancy; the others, promising in a ripening manhood all that a father's heart could desire; both of these went to the field at the first call for troops and both perished in the army.

When such men as yourself and Nott, from the medical profession, and Gen. Wade Hampton, from the head and front of the army—all *representative men*—men who have, in the time of her greatest need, rendered distinguished services to the South, who have been torn and mutilated in person, lacerated and crushed in affections, wrecked and ruined in fortune, can take the proffered hand of friendship and urge conciliation, harmony and fraternization for the good of science and the welfare of the country, I think the personal allusion which I have made to you is pardonable, while it should put to the blush those few "who still urge discord and alienation."

I do not think that a charge of egotism could lie against you in consequence of your publishing what *I think* or say of you. In justice to me you can not omit the reference to you, for, by so doing, you would manifestly defeat one object of the letter. I have seen proper to use your name as a *representative man*, and in a manner to serve a purpose which is obvious throughout the letter, and the *facts warrant the allusion.* I am, dear doctor, very sincerely yours,

W. O. BALDWIN, M.D.

[LETTER II.]

MONTGOMERY, ALA., March 2, 1869.

DR. J. C. NOTT, NEW YORK:

MY DEAR DOCTOR:—As you are aware, the next meeting of the American Medical Association, is to be held in the city of New Orleans, on the first Tuesday in May next, and I write to urge you to be present on that occasion. Your numerous old friends in the South would be most happy to meet you there; to shake you by the hand in this fraternal reunion, and to welcome you again to the scenes of your morning life. It must be gratifying to you to know, my dear, good old friend, when, in your solitary moments, memory sometimes takes you back to the home of your youth, (to review the incidents of almost a life-time spent in active and arduous professional duties,) that your cotemporaries here, who witnessed your devotion to the cause of science, whilst they appreciated the value of your labors, still hold in most affectionate remembrance that honorable courtesy and charity which ever distinguished your conduct towards your professional brothers. I am glad to be able to say, my dear doctor, that the spirit of your example still lives with us, and, I believe, will teach us from the grave; will teach those who still labor in the fields you have left, when life with you shall have ended its hardest lessons. Nothing, I assure you, would give me, individually, more pleasure than to see your honest face on that occasion. It will be such a fitting time for you to meet us, and one which will probably never present itself again, when you could see so many of your old friends.

My correspondence has been somewhat extensive during the past eight or nine months, and I feel justified in saying that the great mass of the profession South is in full accord and sympathy with the Association. You may have seen some little dissatisfaction expressed in newspapers over a *nom de plume*, indicating the author to be a physician, bnt I assure you such sentiments are confined to but very few, and have failed to reach the great heart of the profession. I was grieved, however, to see even this manifestation of opposition to the great representative interests of the medical profession of this country. It has no root and can bear no fruits in science or general beneficence.

This dissatisfaction grew out of the action of the Association at its meeting in 1864, in relation to a preamble and resolutions introduced by Dr. A. K. Gardiner, of New York. These were, in fact, a remonstrance against the war ethics of the Government, and, in substance, provided that the President of the United States, heads of departments and members of the United States Senate be requested by the Associatien to "take such action as shall cause all medicines and medical and surgical instruments and appliances to be excluded from the list 'called contraband of war.'" The action taken on these resolutions by the Association was to lay them on the table indefinitely, and which, in parliamentary *parlance*, I believe, means that it was "not desirable to consider them" at that time. From this action some have contended that the Association lent its influence and support to sustain the Government in this feature of its ethics of war.

The beautiful preamble and resolutions referred to, as having been introduced by Dr. Gardiner, are certainly a most graceful proof of a noble and generous mind, and must be regarded by all as the offspring of the purest and most unselfish charity and benevolence. Yet how far the language used by others in commenting upon this action of the Association is justified by the facts; how far this body lent its influence and support to the Government in the policy complained of, or to what extent it committed itself to the principle, by laying these resolutions on the table, are questions which may very well admit of difference of opinion.

No member can claim for the Association exemption from fair, frank and honorable criticism, and when thus conducted amongst ourselves, or through the legitimate channels of *medical* periodicals, with moderate language and in a courteous and respectful temper, I can see no objection to it, and think it may, in the end, lead to harmony of sentiment and unity of purpose. I have been particularly grieved, however, to see that some, in their zeal to discuss the points above referred to, have resorted to the columns of *newspapers*, (devoted to common and general politics,) for this purpose. The public feel no particular interest in controversies like this, and, in the language of our code of ethics, "as there exists numerous points in medical ethics and etiquette through which the feelings of medical men may be painfully assailed in their intercourse with each other, and which cannot be understood or appreciated by general society, * * * * * * publicity in a case of this nature may be personally injurious to the individuals concerned, and can hardly fail to bring discredit upon the faculty." These injunctions, though applying to our daily intercourse with each other, are equally applicable to us in our associated and general relations.

I am not prepared to say what the usages of modern warfare are on the points raised in Dr. Gardiner's resolutions, or whether there are any recognized or established ethics among civilized nations on this subject. But that it is in accordance with the purest and highest dictates of humanity for belligerent powers to allow the enemy's sick and wounded to be supplied with medicines and surgical appliances from within their own lines, when they cannot be otherwise obtained, I think none will deny, unless the supply be at a time when such action might thwart the movements or prejudice the safety of an army. And, if the duty of regulating such matters had been assigned to the American Medical Association, or even to the army medical corps, and they had established, or advised the establishment of an ordinance making these articles contraband of war, I should feel that their action had not harmonized with the spirit which has ever characterized the conduct of our profession toward suffering humanity.

This, however, was not the case, and I can very well imagine that those who voted against the Association taking the action urged in the preamble and resolutions referred to, could give good reasons which influenced them, at that particular time, to desire no complication with their Government upon a question, in the discussion and decision of which they were regarded as in no way authoritative, and the direction of which had been assumed by high government officials, who had long since estalished and practiced a policy in reference to it.

I assume, then, the broad ground that it was a question with which the Association had nothing whatever to do, and one which was not properly before it for discussion; and, it seems to me that it was expecting too much of our Northern brothers to suppose that they, at a time when all the sinews of war were called most vigorously into execution, would place themselves in antagonism to their government upon a question which was entirely outside of their *professional position and accredited duties.* In doing so, they certainly would have been transcending their legitimate sphere, and meddling with the prerogatives of those to whom the regulation of the ethics of war had been assigned and who claimed exclusive jurisdiction over the question.

Subjects of this kind certainly formed no part in the plan of their organization. They were there solely for the purpose of discussing questions purely scientific and professional, and not such as grow out of civilized warfare. Whatever, therefore, was objectionable in the ordinance alluded to, the high functionaries of the government were alone responsible for it. It was a political and war measure with which the Association had no more to do than did the Pope of Rome or the worshipful grand master of a Masonic Lodge, or any other humane or charitable individual or Christian and benevolent organization in the land. In fact, every man in Christendom was as much to remonstrate with the Government for any violation of the rules of civilized warfare, as were the members of the Association.

It is a very serious and forced conclusion to say, that the Association gave its influence and support to the Government to maintain it in this policy, because it refused at that particular juncture to enter its protest against it, by the the adoption of these resolutions. If, *as an Association*, they had assumed a vindictive or hostile attitude towards the South and advised the adoption of this or any other unjust proceedure on the part of the Northern government, there would have been just reasons for complaint on the part of Southern physicians. This, however, was not the case. The Association simply held itself *firmly* to its *professional position*, to its *acknowledged sphere*, to its *accredited duties*, and refused to go outside of that position to discuss a question which concerned that body no more than it did any private individual in the land.

It is not wise, nor is it required by any creed of general courtesy or ethics, that honor shall always forbid that which honor fails to sanction. Men are not expected or required to denounce every measure of which they cannot approve. There are often good reasons why they should not. Are they then to share the odium of measures entirely foreign to their sphere and beyond their control? There is certainly much difference between the man who commits crime and him who fails to remonstrate with the criminal! As well might we reproach and rebuke the high court of chancery for failing to lecture the world on the subject of religion, the giving of alms to the poor, or for any other philanthropic work which might be calculated to lessen the woes and mitigate the sufferings of fellow beings.

Society, and especially governments, has assigned to different individuals and classes their peculiar sphere and respective duties, and the world owes much of its harmony to this fortunate arrangement. We have our own code of ethics and etiquette, and our own standard of morals, and, if we adhere strictly to these, we cannot interfere with the ethics of war established by ordinances of government. One of the great reconciling principles in the philosophy of life is a proper regard for the rights, duties and principles of others. Whilst, by the very nature of our calling, we are intimately connected with the interests of humanity, and should labor by every means at our command to promote its benefactions, we must be careful in our zeal for a good cause not to hazard the position and influence already gained, by invading the precincts and prerogatives of others.

The restraints and usages of governments in times of war may seem to us, in many particulars, unnecessarily harsh, oppressive and cruel, and, indeed, what civilian ever witnessed the operation of martial law who could not find grave objections, both to its humanity and equity? But when these have been ordained by persons to whom we are only subordinate, we cannot be responsible for results, and should, in no way, share the odium, simply by failing to place ourselves in open antagonism to them.

As long as we labor with all the professional, intellectual and moral efficiency at our command, for the fulfillment of duties properly within our legitimate and recognized sphere, we shall have accomplished all the good for humanity that the world can reasonebly expect or require of us.

But even suppose the Association did commit an error, in fact and in spirit, in failing to remonstrate with its government as stated, where is the wisdom, at this day, of opposition to its future and permanent interests? Suppose that the feeble assaults which have been made upon it should swell into an hostility whose magnitude should in the end mar its progress, compass its disorganization and defeat its claims to a grand nationality, who could receive credit for such a work? Where would be the glory of success or the fruit of such victory? Could science, could humanity, could the country thank one for such a service?

What has brought the science of medicine to its present state of advancement but the labor of *intellects combined in organization?* Like the tiny insect which lays up its stores for the wants of winter, we, too, must acknowledge the great law which sanctions the wisdom of associated labor. The imperishable grandeur and usefulness of all sciences owe their highest development to organized effort. The future glories of the science of medicine in this country, lie embodied in the powers yet latent in organization, and he who seeks to disturb this great element in its prosperity is no friend to progress.

The animus of the Association has shown itself to be honorable and kind in every reference made to its Southern members, during and since the war; honorable to itself, honorable to the profession, honorable, just and generous to the South. When I went to its last meeting (in Washington) I did so from a sense of duty, and with the earnest desire of seeing the two sections united in their professional relations and purposes. I did not solicit any honors, and asked no man to vote for me for any office. Yet with a meagre representation from the South, they conferred upon me the highest office in their gift. I knew myself unworthy of the high distinction, and felt it was not intended for me. I knew it had a broader and higher significance than that of a mere tribute to personal and private ambition. I knew it to be in keeping with that kindly spirit displayed by the Northern delegates towards their Southern brethren throughout their "Transactions," and that it was but a fresh offering of the olive branch of peace. In this spirit I

accepted it. No man asked me any thing in relation to my political sentiments. I cannot boast of performances in the late struggle, but I have never disguised the fact from any one, that in all the earnest desires of the heart which constitute devotion to a cause, I yield to none in my loyalty to that which has gone down in the gloom of defeat and for which those tender youths, your son and mine, fought side by side, and fighting fell for principles held dear by you and me. I would not stultify myself on this point for all the honors which could be heaped upon me by the medical profession or any other class of men. Nor do I think my Northern brothers would respect me more for being false to my section. In the death of my boy I found the hardest heart-sorrow of my life, and the weary years which have since passed by have been powerless to still its anguish; and yet I could but feel a mournful pride in a knowledge of the fact, that he died on the field of glory and *true to the land which gave him birth.*

But the crushed affections and blighted hopes of the father, who has yielded a noble sacrifice to his country, as he sits in silent and sacred memory of his holiest grief, can find no relief by barbing the anguish of his heart with feelings of malice, hatred and revenge towards those who, in honorable combat, had been made the instruments of his sorrow. Natural affection does not require this; true manliness does not demand it.

No, Doctor, I do not wish to cherish feelings of bitterness with the memory of my son. I wish to forget all that is painful and harrowing to the heart, and to remember him as he was, the soldier, patriot and Christian, falling in honorable warfare, and, that the hand which sent the fatal ball which deprived him of life, was that of some brave and generous spirit, moved by the same high purpose, the same stern sense of duty, the same devotion to principle and country, which guided and actuated him. So far from entertaining sentiments of unkindness towards our brothers of the medical profession North, growing out of this affliction, my only feeling has been, that if any one of them had been near him in that dreadful hour, his highest care would have been to have drawn, if possible, the fatal ball from his breast, and restored him to life and health.

How unwise and unprofitable it is to seek to mingle the temper of partizan strife with the affairs of a great science. If the gallant General Hampton, whose blood flowed so freely in the late war, and whose home, with the homes of his people, was consumed and made desolate by the flames of the Northern army, can speak gratefully of "the spirit of conciliation, the magnanimity and the kindness" of those "who recognize us as no longer foes but brethren," can, for his country's good, declare his willingness to bury "all past differences in one common grave," to "accept the right hand of fellowship * * * so frankly extended," and greet, as a "comrade," him whose hand "so lately grasped the sword," but now bears the olive branch of peace," shall we be so sectional and prejudiced as to nurse feelings of hostility towards a brotherhood, from whom we have ever received only evidences of marked kindness and honorable courtesy?

If the talented and independent editor of the Richmond and Louisville Medical Journal, Prof. E. S. Gaillard, who lost his right arm, when a medical director, in the discharge of surgical duties on the field of battle, thus depriving him of all hope of further advancement in the special department which had been the choice of his youth, for which genius, education and a thorough method had so well prepared him, and to which the achievements of early manhood had already given such brilliant promise of successful ambition; I say if he can advise that we should cover over the past "with the mantle of personal and professional charity," that we should "take the out-stretched hand, accept the offer of friendliness and reconciliation;" and that the reception of the "*medical men of America*," when they assemble in New Orleans, in May next, should be "not only a hospitable reception, but a warm, a manly and a generous welcome," cannot those who never felt a wound, and can even jest at scars, lay aside feelings which can neither yield fruits to our noble science nor do honor to our manhood? Is any one vain or weak enough to believe that our Northern brothers will derive an advantage from fellowship, union and harmony which we will not share in an equal ratio?

Pardon me, dear doctor, for trespassing so long upon your valuable time. I know you will excuse it in the interest which you feel in the general prosperity of the medical profession of the whole country, and especially in the desire which you feel to see your Southern friends come fully up to their duty, in meeting the honorable advances which have been made by our Nothern brothers, looking to a complete and perfect fraternization. I think the

American Medical Association is to be the power through which a greater good is to be accomplished for the profession in this country, than has yet been achieved. On this point you may, perhaps, hear from me at some future time. I will only say now, that its organization had its inception chiefly in an idea which has not yet been realized—that of elevating the standard of medical education in this country. But I believe its labors in this direction will yet be felt and acknowledged. To this end *it must be national* and represent the interests of the profession in every part of the the country. Those who comprehend the grandeur of its germ, appreciate full well the ultimate possibility of its nature, and will see to it, that the inspiration which gave it birth shall be worked to a successful end. The advancement of science, the affections of an enlightened brotherhood, the interests of society and the good of humanity are all united with it, and from every section I have the most gratifying assurances of a determination to bury all other sentiments in the one great purpose of promoting harmony and concert of action, with the kindest feelings of fraternal regard. Assure our friends of the North of this, and tell them we desire to meet them in large numbers in New Orleans in May.

With assurances of the highest regard, believe me, dear doctor, most sincerely and truly your friend,

W. O. Baldwin, M. D.

[LETTER III.]

New York, No. 4 West Twenty-Second Street,
March 8, 1869.

W. O. Baldwin, M.D.:

My Dear Doctor:—Your letter of the 2d has just come to hand. I hasten to reply by return mail.

Whilst I am fully sensible that your kind feelings for me have tempted you to speak in terms of praise beyond my merits, I have the vanity to believe that you do not over-estimate my high sense of obligation to our noble profession; my unceasing efforts to uphold its dignity, and my endeavors to promote friendly feelings amongst its members. I have always maintained that we could not deserve or command the respect of the world, unless we respected each other, and preserved a proper *esprit de corps*.

When I was about to take my farewell of the people of Mobile, among whom I had lived for thirty years, the leading citizens gave me a public dinner, and the members of the profession a handsome reception, at which I was presented with a piece of plate, on which was engraved the name of every regular practitioner of the city. This, to me, was the crowning glory of a long career, as it was the grateful evidence to me that my constant efforts to keep the members of the profession together in brotherly love and usefulness had not been in vain.

You may well believe then, my dear friend, that your present efforts in the same good cause, on a wider field, meet my hearty approbation and sympathy. I have nothing to suggest in addition to your excellent letter, which covers the whole ground at issue; it is temperate, honest, manly, and in every way becoming the high and responsible position in which you are placed. I doubt not it will be responded to by the profession North, South, East and West, in the same spirit in which it was conceived.

The construction you have given to the action of the American Medical Association, on the premable and resolutions of Dr. A. K. Gardner, to which you refer, corresponds precisely with what I have heard expressed by all the members of the profession I have met at the North. The time of the Association was fully occupied with matters that properly belonged to it, and these resolutions, trenched upon political or military considerations which were foreign to the business of the Association, which they could not influence. Any debate upon them might have led to some unpleasant remarks from some impetuous member, and it was therefore, best to lay them on the table. If such resolutions had been laid before any hundred members of our profession, during the war, at the South, what, let me ask you, would have been the result? There is a statistical law that throws a certain per cent. of unwise heads into every assembly of this kind, and the less opportunity they have of talking, the better.

Now, sir, I beg leave to say a word of my personal experience, since the war at the North. Soon after the was was closed, I was summoned to Washington as a witness in the Wirz trial, and seized the occasion to run over to Philadelphia to see what I could discover that was new, in the way of books, instruments, practice, etc., we having been shut out from the world for four years. Not only did the medical gentlemen of Philadelphia receive me politely, but they seemed to feel as if they thought I might feel some delicacy in presenting my rebel face in their midst, and were more desirous, than I had ever seen them, of treating me with hospitality.

About a year ago I came to pitch my tent in the city of New York, determined to ask no favors of the members of the profession, and not one of them can say that I ever solicited an introduction to him; and yet it would seem like egotism were I to tell of half the respect, the hospitality and kindness I have received, both in and out of the profession in the city of New York. It is but justice to the faculty in New York to say that in tone, talent and attainment, it will compare favorably with that of the large capitals of Europe.

But, suppose we admit that the action of the Association on the resolutions of Dr. Gardiner was dictated by sectional and unchristianlike motives, this does not alter the case. The war is over; our prosperity and happiness depend upon our return to the former status of the country, politically and socially; passion and prejudice should be laid in the grave with the half million of brave men that have been buried in the bloody strife. The olive branch has been gracefully and cordially tendered by our medical brethren at the North to those at the South, and it is your duty to accept it frankly and in good faith.

The medical profession has a great mission to fulfill. Medicine is not only a healing art, but is the mother of anatomy and physiology in their most extended sense; of botany, chemistry, mineralogy, geology, etc., in fact, of all the natural sciences, from which have sprung the useful arts. It has been the great fountain, from which has flowed the elements of civilization, from the foundation of the Egyptian empire to the present day.

Now, my dear friend, will the medical profession at the South be outdone in magnanimity? Will they permit a petty pique, or even the remembrance of a great civil war, in which, perhaps, we were all to blame, to cross the path of science, and to mar a great enterprise like that of the Medical Profession?

God forbid! My many old friends must throw aside all minor considerations and come forward in sustaining your efforts to maintain the true honor of the South, the dignity of our profession and the cause of humanity.

Very truly, your friend,

J. C. Nott.

